# Bioactive Hydrogels and Scaffolds for Oral Mucosal Regeneration After Oral Squamous Cell Carcinoma Therapy: A Comprehensive Review

**DOI:** 10.3390/medicina62030558

**Published:** 2026-03-17

**Authors:** Alina Ormenisan, Andreea Bors, Liana Beresescu, Despina Luciana Bereczki-Temistocle, Gabriela Felicia Beresescu

**Affiliations:** 1 Department of Oral and Maxillo-Facial Surgery, George Emil Palade University of Medicine, Pharmacy, Science, and Technology of Târgu Mureș, 540139 Târgu Mureș, Romania; alina.ormenisan@umfst.ro; 2 Department of Tooth and Dental Arch Morphology, George Emil Palade University of Medicine, Pharmacy, Science, and Technology of Târgu Mureș, 540139 Târgu Mureș, Romania; andreea.bors@umfst.ro (A.B.); felicia.beresescu@umfst.ro (G.F.B.); 3 Department of Preventive and Community Dentistry, George Emil Palade University of Medicine, Pharmacy, Science, and Technology of Târgu Mureș, 540139 Târgu Mureș, Romania; liana.beresescu@umfst.ro

**Keywords:** hydrogel, oral squamous cell carcinoma, scaffold, tissue engineering, mucoadhesion, biomaterials

## Abstract

Oral squamous cell carcinoma (OSCC) therapy frequently produces acute and chronic injury to the oral mucosa, including surgical lining defects and radiochemotherapy-associated oral mucositis (OM). Beyond pain and ulceration, these injuries compromise nutrition, speech, oral hygiene, and feasibility of dental/implant rehabilitation, and may disrupt oncologic treatment delivery. The oral cavity imposes stringent constraints on regenerative biomaterials—continuous salivary flow, high microbial load, and repeated mechanical shear—such that clinical success depends on reliable mucoadhesion/wet adhesion, barrier function, mechanical compliance, and safe, spatially confined bioactivity. This PRISMA-informed evidence-mapped structured narrative review provides an evidence map and structured qualitative synthesis of hydrogel and scaffold platforms relevant to post-OSCC care, spanning clinically used mucoadhesive barrier formulations through emerging wet-adhesive multifunctional patches, acellular matrices, and tissue-engineered oral mucosa (TEOM) constructs. Clinically, the strongest evidence base remains barrier-forming gels and liquids that reduce OM pain and improve oral function during active therapy, establishing performance benchmarks for intraoral retention and patient-reported benefit. Preclinical studies are rapidly expanding toward multifunctional designs that integrate antimicrobial, anti-inflammatory, pro-epithelialization, and pro-angiogenic cues. However, a pervasive limitation is the inconsistent use of OSCC-relevant models (e.g., irradiated/xerostomic tissue beds), standardized functional endpoints (e.g., oral intake, durability under mastication, and neurosensory outcomes), and explicit oncologic safety evaluation, which severely compromises translational validity. For reconstructive applications, dermal matrices and early TEOM reports suggest feasibility for selected defects, but controlled comparative trials and scalable manufacturing pathways remain limited. Translational priorities include oncologic-by-design bioactivity (time-limited, locally confined cues), clinically anchored outcome reporting, and quality-by-design manufacturing aligned with device/combination/advanced-therapy regulatory requirements.

## 1. Introduction

Oral squamous cell carcinoma (OSCC) remains a leading cause of morbidity and mortality within head and neck oncology and is associated with substantial long-term functional disability [1,2]. For resectable disease, primary surgery is standard, with adjuvant radiotherapy or chemoradiotherapy recommended for patients with higher-risk pathologic features [2,3,4,5,6]. While oncologic outcomes have improved, treatment commonly results in clinically consequential oral soft-tissue injury, surgery-associated lining defects, and therapy-associated oral mucositis (OM), creating a survivorship burden that directly impairs eating, speaking, oral hygiene, and subsequent dental/implant rehabilitation and can also necessitate treatment interruptions or dose modification [4,7,8].

The oral mucosa is a uniquely demanding target for regeneration because barrier integrity must be maintained under continuous salivary flow, a dense and dynamic microbiome, and repeated mechanical loading during mastication and speech [9]. Following OSCC therapy, multiple layers of tissue organization are disrupted, including epithelial continuity, basement membrane integrity, lamina propria architecture, microvascular supply, salivary gland function, and neurosensory pathways. Radiotherapy further drives microvascular injury, chronic inflammatory dysregulation, and fibrotic remodeling that collectively reduce tissue compliance and impair re-epithelialization, while xerostomia and microbial dysbiosis amplify irritation, infection susceptibility, and delayed healing [7,10,11]. Consequently, meaningful oral soft-tissue regeneration must be evaluated not only by macroscopic closure or histology but also by durable function in a wet, mechanically active, microbially rich environment.

Acute oral mucositis (OM) remains one of the most frequent and debilitating toxicities of radiotherapy and chemoradiotherapy in head and neck cancer [9,10,11]. OM progresses through a multi-stage cascade involving epithelial/submucosal injury, reactive oxygen species generation, inflammatory amplification, ulceration, and secondary microbial colonization, followed by a prolonged healing phase that can extend beyond active treatment [12,13,14]. Most current interventions focus on prevention and symptom control, often reducing severity and pain but not fully restoring mucosal architecture or preventing late sequelae [11]. Moreover, late radiation effects, xerostomia, scarring, and impaired remodeling create a chronic vulnerability wherein minor trauma can precipitate recurrent ulceration and soft-tissue breakdown [4,7,8,10]. These realities motivate regenerative strategies that can rapidly re-establish barrier function while improving tissue quality (organized epithelium, controlled inflammation, reduced infection and contracture, and better functional durability).

Bioactive hydrogels and scaffolds have therefore emerged as a continuum of solutions bridging symptom-oriented barrier dressings and complex cell-based grafts. Hydrogels can conform to irregular intraoral wounds, provide mucoadhesion or wet-tissue adhesion, reduce frictional trauma, and locally deliver analgesic, anti-inflammatory, antimicrobial, or pro-regenerative cues [15,16]. Porous scaffolds and hybrid constructs can add mechanical stability and spatial guidance for larger lining defects, and Tissue-engineered oral mucosa (TEOM) systems aim to recapitulate epithelial stratification on ECM-like stromal matrices [15,16,17,18]. Importantly, regeneration in OSCC survivorship must be pursued with explicit oncologic safety constraints, because regenerative cues may intersect with tumor-supportive stromal programs within the cancerized and therapy-altered field [19,20].

This review first delineates the post-OSCC therapy microenvironment and functional design requirements, then critically appraises hydrogel and scaffold platforms (including TEOM strategies), and finally discusses translational readiness, oncologic safety, neurosensory considerations, and regulatory, scalability, and cost challenges.

## 2. Materials and Methods

### 2.1. Review Approach and Reporting Framework

Because oral mucosal biomaterial research is heterogeneous in terms of materials, delivery formats, model systems, and outcome reporting, this work was conducted as an evidence-mapped narrative review using a structured qualitative (framework-based) synthesis. Reporting was aligned with PRISMA 2020 guidance where applicable to enhance transparency and reproducibility (see Supplementary Materials) [21].

A protocol was not preregistered. Searches were conducted from database inception to the final search date and were limited to full texts available in English.

**Figure 1:**
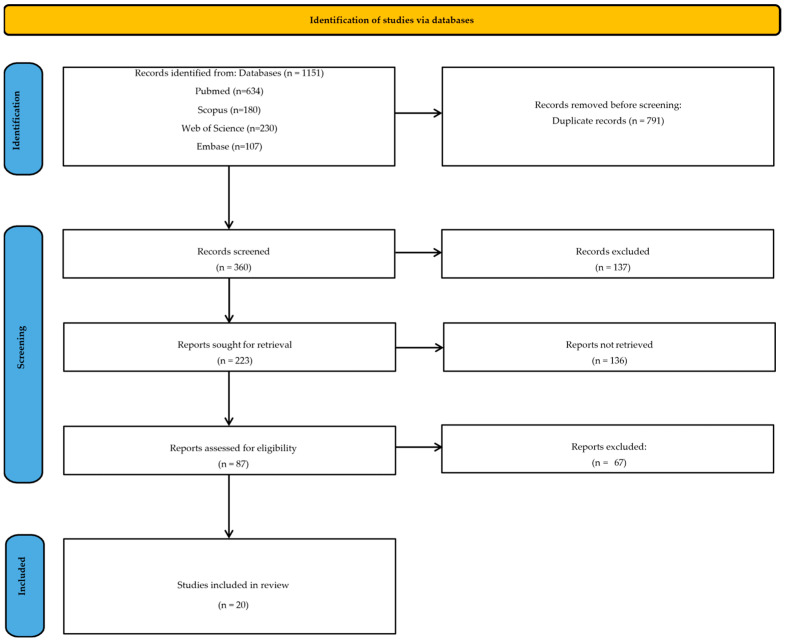
PRISMA flow diagram (study selection).

Study identification, screening, eligibility, and inclusion are summarized in the PRISMA flow diagram (Figure 1).

### 2.2. Information Sources

To capture both biomedical and biomaterials engineering evidence relevant to oral mucosal regeneration after OSCC therapy, the following databases were searched: MEDLINE (via PubMed), Embase, Web of Science, and Scopus. Backward and forward citation tracking of key reviews and seminal primary studies was used to reduce database indexing bias and capture influential studies not retrieved by keyword strategies.

### 2.3. Search Strategy

The electronic search strategy was constructed using three concept blocks: (i) oral mucosa and mucosal wound-healing phenotypes, (ii) biomaterial platforms and tissue-engineering constructs, and (iii) OSCC treatment context and therapy-associated injuries (OSCC/post-therapy oral mucosal injury was the primary scope). Controlled vocabulary (e.g., MeSH in MEDLINE and equivalent Embase indexing) was combined with free-text keywords to accommodate variable indexing and emerging terminology. The PubMed/MEDLINE strategy used terms including Mouth Mucosa, oral mucosa/buccal mucosa/oral wound/mucositis/ulcer*, combined with hydrogel*/scaffold*/biomaterial*/tissueengineering/extracellularmatrix/bioprint*/electr0spin*/wound dressing, and combined with OSCC/head and neck/radiotherap*/chemoradiotherap*/radiation injury/postoperative.

Detailed database-specific search strategies and search dates for each database (adapted for MEDLINE, Embase, Web of Science, and Scopus) are consistent with the PRISMA Search Strategy extension [21].

### 2.4. Eligibility Criteria

Evidence was eligible if it addressed at least one of the following domains in an oral mucosal context relevant to OSCC patients:

Inclusion criteria

- Bioactive hydrogels, porous scaffolds, hybrid materials, or TEOM constructed and designed to promote oral mucosal repair/regeneration (re-epithelialization and/or restoration of supporting soft tissues).
- Models directly relevant to post-OSCC therapy environments, including post-resection mucosal defects, irradiated or chemoradiated tissue beds, and models of radiation-associated mucositis/fibrosis.
- Evidence spanning in vitro (e.g., epithelial adhesion/stratification), in vivo animal studies, and human studies (case series, early-phase trials, comparative cohorts).
- Reviews/consensus statements were used for contextualization (mechanisms, outcome measures, clinical comparators) but were not treated as primary evidence.

Exclusion criteria

- Platforms developed primarily for anti-tumor drug delivery without explicit regeneration or wound-healing endpoints.
- Studies focused exclusively on hard-tissue regeneration (bone/dentin/enamel), unless a distinct oral mucosal component directly relevant to this scope was included.
- Cutaneous/non-oral mucosal models without a clear mechanistic or design-transfer rationale to the oral environment.

### 2.5. Study Selection and Data Extraction

Study selection was performed in two stages: (i) title/abstract screening and (ii) full-text eligibility assessment. Duplicates were removed prior to screening using reference management software. Two reviewers screened records and resolved disagreements by consensus. The PRISMA diagram (Figure 1) reports the identification and screening outcomes.

For included studies, data were extracted using a standardized charting approach, including:

- Material composition and chemistry (natural/synthetic/hybrid; crosslinking modality; degradation mechanism).
- Architecture and mechanics (porosity/microstructure; modulus/viscoelastic metrics; swelling; water content).
- Form factor and application route (rinses; in situ gelling systems; mucoadhesive patches/films; sponges/membranes; 3D-printed constructs).
- Bioactivity strategy (ECM-mimetic motifs; growth factors/cytokines; antimicrobials; immunomodulators; release strategy).
- Cellular components where applicable (cell type; autologous/allogeneic; culture/maturation conditions).
- Model characteristics (species; oral site; wound type; irradiation/chemotherapy exposure; follow-up).
- Outcome measures (time to closure; histology; angiogenesis; inflammatory markers; microbial burden; fibrosis/contracture; adverse events).
- Oncologic safety reporting (e.g., dysplasia assessment, recurrence surveillance, and evaluation of tumor-promoting signaling in response to delivered cues).

Given heterogeneity of study designs and endpoints, the risk of bias was appraised using design-specific tools such as the Cochrane Risk of Bias tool (RoB 2.0) for randomized controlled trials and ROBINS-I for non-randomized studies. The strength of evidence was graded using a modified hierarchy of evidence based on study design and methodological quality: Level I: Randomized controlled clinical trials; Level II: Non-randomized clinical trials or well-designed cohort studies; Level III: Animal studies with validated models; Level IV: Mechanistic in vitro investigations. In order to enable the identification of research gaps and assessment of translational potential, evidence was stratified according to: study type (clinical vs. preclinical), biomaterial class (hydrogels, mucoadhesive, scaffolds, drug-delivery systems), OSCC-relevant microenvironmental conditions, and reported therapeutic outcomes. For preclinical animal studies, internal validity was assessed qualitatively due to widespread incomplete reporting, as a formal systematic risk-of-bias tool application was often hindered by insufficient detail. This methodological constraint, inherent to the current preclinical landscape, means that while PRISMA guided our evidence mapping, a full systematic quantitative risk-of-bias assessment was not feasible for all included studies, particularly those in early preclinical development.

### 2.6. Evidence Synthesis and Analytical Framework

Due to the profound heterogeneity in material compositions, model systems, and outcome reporting across studies, a formal meta-analysis was not feasible. Therefore, synthesis was performed as a structured narrative qualitative synthesis, organized into practical taxonomies reflecting clinical decision points. While a purely quantitative synthesis was not appropriate, we aimed to systematically interpret evidence by stratifying findings. Evidence was stratified by: (i) material class (natural, synthetic, hybrid/composite), (ii) delivery format (rinses/in situ gels, mucoadhesive patches/films, porous scaffolds, TEOM constructs), and (iii) intended bioactive function (pro-epithelialization, pro-angiogenic, immunomodulatory/anti-inflammatory, antimicrobial, antifibrotic/anti-contracture).

Within each stratum, studies were interpreted through a translational readiness lens emphasizing fabrication reproducibility, sterilization/storage feasibility, manufacturability, safety in irradiated tissue beds, and alignment with clinically meaningful endpoints (e.g., reduction in wound complications, faster return to oral intake, improved pain control, durable barrier restoration). Approaches explicitly incorporating OSCC-relevant constraints (irradiation, microbial dysbiosis, xerostomia, cancerization-field considerations) were prioritized because these factors materially influence performance and risk–benefit assessments in survivorship care.

## 3. Results

Research on oral mucosal regeneration after OSCC therapy spans (i) clinically used mucoadhesive barrier products for OM symptom mitigation, (ii) scaffold/matrix approaches for post-resection lining defects, and (iii) a rapidly expanding preclinical pipeline of wet-adhesive multifunctional patches and TEOM-enabling scaffolds (Table 1).

### 3.1. Post-OSCC Therapy Microenvironment and Barriers to Regeneration

OSCC treatment commonly combines surgical resection with radiotherapy and, in selected cases, concurrent systemic therapy. Surgery produces lining defects that require rapid re-epithelialization to restore barrier function and permit oral intake; in severe contexts, barrier loss and pain can contribute to nutritional compromise and feeding tube dependence [22,23]. Radiotherapy/chemoradiotherapy introduces additional constraints, including acute OM with erythema and ulceration, microbial translocation, and secondary infection, as well as chronic vascular rarefaction, salivary gland hypofunction, persistent inflammatory signaling, and fibrosis. These changes impair granulation tissue quality, epithelial migration, and remodeling, increasing the risk of chronic ulceration, soft-tissue breakdown, and functional deficits. Reviews and consensus statements consistently identify OM as a dose-limiting toxicity and emphasize supportive care focused on pain control, nutrition maintenance, and infection prevention [24,25,26].

Collectively, the literature supports that biomaterials for post-OSCC oral mucosal repair must function within a hostile microenvironment characterized by persistent inflammation, microbial dysbiosis, mechanical stress, and the need for oncologic safety [27]. These constraints shape design requirements that extend beyond simple “wound coverage” toward durable retention, microbial control, controlled and spatially confined bioactivity, and comfort under oral function.

### 3.2. Functional Design Requirements for Intraoral Hydrogels and Scaffolds

Across platforms, the dominant functional requirement is retention on wet mucosa under salivary flow and shear. Intraoral biomaterials commonly rely on the following: (i) mucoadhesion (electrostatic interactions, hydrogen bonding, chain interpenetration), (ii) wet-tissue adhesion (catechol chemistry, ionic complexes, dynamic covalent bonds), and/or (iii) mechanical anchoring via microstructured patches or interlocking networks [15,16,28]. For OSCC survivors, equally critical secondary requirements include antimicrobial barrier activity, hemostasis/exudate management in surgical beds, analgesic and anti-inflammatory effects, and controlled degradation that avoids premature loss or prolonged foreign-body responses [11,15,17,18,19,29,30]. Contemporary guidance further emphasizes that clinically meaningful benefit is often reflected in functional outcomes (oral intake, opioid use, treatment continuity) rather than mucosal appearance alone [31,32,33,34].

### 3.3. Hydrogel Platforms for Oral Mucosal Wound Coverage, Drug Delivery, and Regeneration

Hydrogels are widely investigated in oral regenerative medicine because they can be engineered as rinses, sprays, injectables, in situ-forming gels, or preformed patches. They provide moist wound coverage, reduce frictional trauma, and serve as local depots for therapeutic payloads [35,36]. 

In OSCC survivorship, the translation pathway often progresses from (i) symptom-focused barrier hydrogels for OM and ulcerated mucosa toward (ii) more explicitly regenerative hydrogels incorporating cues that accelerate re-epithelialization and improve tissue quality [37,38].

#### 3.3.1. Symptom Control Versus Regeneration: Clinical Benchmarks and Operational Definitions

In this review, interventions were classified as primarily symptom-oriented when their dominant demonstrated effect was barrier/lubrication and short-term symptom relief (e.g., pain reduction) without evidence of durable tissue restoration. Interventions were classified as regenerative when they met one or more of the following operational criteria:

- Evidence of organized epithelial stratification and phenotypic maturation (beyond simple re-epithelialization);
- Sustained functional restitution (e.g., durable barrier performance under oral loading);
- Demonstrated modulation of fibrosis/contracture or stromal remodelling relevant to post-therapy defects;
- Evidence of architectural restoration (epithelium + supportive stroma/ECM organization), not only faster closure.

Mucoadhesive hydrogel rinses (e.g., MuGard) provide evidence for symptom mitigation in randomized placebo-controlled settings but focus mainly on acute OM symptoms rather than long-term tissue restoration [39]. Bioadhesive barrier-forming oral liquids (e.g., CAM2028/Episil) have demonstrated rapid pain reduction in randomized multicenter studies, and additional studies in radiotherapy contexts suggest potential improvements in OM burden and nutritional status [37,40]. While these products are not regenerative matrices per se, they establish pragmatic performance benchmarks for next-generation materials—rapid application, high intraoral retention, and demonstrable patient-reported benefits, such as ease of application, comfort during wear, taste, and impact on daily activities, which are crucial for patient adherence and overall treatment success. 

#### 3.3.2. Material Families and Crosslinking Strategies

Natural polymers (e.g., collagen/gelatin, hyaluronic acid, chitosan, alginate) remain attractive due to inherent biocompatibility and cell-instructive motifs. Oral regenerative literature frequently employs these materials alone or in composites to address shortcomings such as rapid degradation, limited mechanical strength, or insufficient adhesion [15,16]. Clinically, hyaluronic-acid formulations have been explored for improving the healing of oral cavity surgical incisions [41]. Collagen/gelatin systems can be crosslinked physically (temperature/ionic interactions), chemically (e.g., carbodiimide, genipin), or via photocrosslinkable derivatives (e.g., GelMA) to tune mechanics and degradation [18,42].

Synthetic and semi-synthetic polymers (e.g., PEG-based networks, poly(vinyl alcohol), poloxamers) offer batch consistency and design flexibility but often require bioactive ligands to support adhesion and remodeling. Hybrid hydrogels are increasingly favored for intraoral applications because they can couple strong adhesion and mechanical robustness with controlled delivery of therapeutic payloads [15,16].

#### 3.3.3. Wet-Adhesive and Multifunctional Hydrogel Patches

Because the oral cavity is persistently wet and mechanically active, recent innovations emphasize wet-adhesive patches that maintain intimate contact with the wound bed. A representative example is a hydrogel adhesive based on a photo-triggered S-nitrosylation coupling reaction reported to promote oral mucosal wound healing in vivo [43,44]. Related designs integrate antimicrobial barrier functions and high retention—e.g., wet-adhesive poly(ionic liquid) patches with antibacterial and anti-inflammatory activity in infected oral ulcer models [45], moisture-responsive bioadhesive patches designed to improve placement and retention [46], and Janus hydrogel patches combining a wet-adhesive surface with a protective barrier layer to enhance microbial control and regeneration in oral ulcer models [22]. This platform was evaluated in non-irradiated acute ulcer models; therefore, extrapolation to OSCC survivorship beds should be considered with caution, given the absence of irradiation, xerostomia, and dysbiosis.

For OSCC patients, these patch architectures remain conceptually important even when not OSCC-specific, because they demonstrate how modern hydrogel design can simultaneously address retention, microbial control, and tissue repair. Translational relevance depends on pairing these architectures with oncologically appropriate, time-limited bioactivity and testing in OSCC-relevant tissue contexts (e.g., irradiated beds) [47,48].

### 3.4. Scaffold-Based Constructs and Tissue-Engineered Oral Mucosa

Scaffolds provide structural guidance that hydrogels alone may not achieve, particularly for larger lining defects requiring mechanical integrity and resistance to contraction. In oral soft-tissue engineering, scaffolds are often fabricated as porous sponges, membranes, or bilayer constructs and used either acellularly (host-cell recruitment) or as substrates for pre-seeded epithelial and stromal cells (TEOM) [15,16,17,18].

#### 3.4.1. Acellular Matrices and Dermal Substitutes for Oral Resurfacing

Decellularized matrices aim to preserve ECM architecture and biochemical cues while minimizing immunogenic cellular content. ECM-based scaffolds can support cellular infiltration and constructive remodeling; however, outcomes depend on decellularization quality, sterilization, and the recipient bed condition [49,50]. In the intraoral setting, regenerative dermal matrices have been reported for resurfacing mucosal defects after resection of early-stage oral carcinomas with the goal of reducing donor-site morbidity relative to autologous grafts [51,52]. Evidence remains largely limited to case series and institutional experiences, highlighting the need to better define indications, fixation strategies, infection risk in the oral microbiome, contraction risk, and long-term functional durability. Next-generation designs should explicitly integrate robust fixation/adhesion strategies, antimicrobial features, and mechanics tailored to intraoral loading.

#### 3.4.2. Tissue-Engineered Oral Mucosa Equivalents

TEOM constructs typically combine autologous oral keratinocytes with stromal matrices that support stratification at the air–liquid interface. Early work demonstrated serum-free fabrication and preliminary clinical grafting of ex vivo-produced oral mucosa equivalents, with subsequent reports exploring intraoral lining reconstruction and immunohistological assessment of phenotype and engraftment [53,54,55].

From a biomaterials perspective, scaffold selection strongly influences mechanical handling, epithelial maturation, and durability. Collagen-based full-thickness oral mucosa equivalents have been developed to recapitulate epithelial and connective tissue compartments [56], and composite scaffolds (e.g., chitosan–collagen) have been used to improve mechanics and modulate degradation while supporting epithelial differentiation [57]. These approaches align with OSCC reconstruction needs, where success requires not only rapid coverage but also a durable mucosa that resists breakdown under mastication and in irradiated fields.

In total, nine clinical studies (four randomized and five non-randomized), four animal studies, and three in vitro investigations met the inclusion criteria (Table 2). Among preclinical in vivo studies, 0% (0/4) used OSCC-relevant models (e.g., irradiation/xerostomia/dysbiosis), whereas 100% (4/4) relied on acute oral ulcer/defect models in otherwise healthy animals.

Table 3 highlights examples of clinically oriented hydrogel and scaffold biomaterials investigated for restoring oral mucosal tissue following therapeutic interventions and surgical resection in head and neck cancer.

### 3.5. Translational Readiness and Clinical Evidence

While mucoadhesive barrier-forming gels and liquids represent the most clinically mature interventions for alleviating OM pain and improving oral function, the evidence base for true, durable regeneration and long-term functional restoration remains nascent and largely stems from studies with a higher risk of bias. Among clinical studies, n_1_ = 7 (≈77.8%) were conducted specifically in head and neck cancer or OSCC-post-therapy settings, while n_2_ = 2 (≈22.2%) were extrapolated from broader oral ulcer or non-oncologic oral surgery populations. This underscores the critical need for well-designed, adequately powered clinical trials focused on comprehensive, long-term functional endpoints [37,39,40]. For surgical reconstruction, the most practical scaffold-based options currently include acellular dermal/regenerative matrices for selected mucosal defects, potentially reducing donor-site morbidity [51,52]. TEOM approaches offer a more biomimetic strategy but remain constrained by scalability, manufacturing complexity, cost, and regulatory pathway requirements typical of advanced therapies [53,54,55,56,57]. Existing clinical reports are largely proof-of-concept with small cohorts and limited follow-up, underscoring the urgent need for well-designed, adequately powered randomized controlled trials to establish efficacy and safety, thereby forging clearer translational pathways.

### 3.6. Oncologic Safety Considerations in OSCC Patients

Unlike many benign oral wounds, OSCC-related defects occur within a cancerized field characterized by chronic inflammation and ongoing recurrence surveillance. A central translational concern is that regenerative signals, including pro-angiogenic/pro-proliferative growth factors, immunomodulators, and transplanted stromal cells, could, under some conditions, support residual malignant cells or create a permissive microenvironment. The conceptual relationship between wound healing and tumor stroma (“tumors as wounds that do not heal”) is well recognized [20], and contemporary analyses highlight overlaps between regenerative remodeling programs and tumor-supportive stromal states [19]. Caution is warranted for pathways implicated in tumor progression (e.g., TGF-β and HIF-1α signaling) [22,58].

Accordingly, biomaterials intended for OSCC patients should be designed with a “oncology-by-design” approach: (i) prioritize short-acting, locally confined cues rather than sustained exposure to potent mitogens; (ii) emphasize barrier restoration, microbial control, and inflammation modulation that reduces chronic tissue damage; (iii) incorporate in vitro screening against OSCC-relevant cell lines and co-culture models; and (iv) evaluate performance in models that better approximate survivorship constraints, including irradiated tissue beds and, where feasible, residual-disease proxies. These considerations are particularly important for cell-based therapies and long-persisting scaffolds in post-treatment tissues.

Three recurring gaps remain clear. A pervasive and significant translational gap observed across the preclinical evidence is the widespread reliance on acute oral ulcer models in healthy animals, which fundamentally misrepresents the post-OSCC microenvironment characterized by irradiated tissues, xerostomia, dysbiosis, and repeated mechanical trauma. Of the 4 preclinical in vivo studies included, only 0 (0%) used irradiated or otherwise OSCC-relevant tissue beds, whereas the remaining 4 (100%) relied on acute ulcer models in non-irradiated animals. This platform was evaluated in non-irradiated acute ulcer models; therefore, extrapolation to OSCC survivorship beds should be considered with caution, given the absence of irradiation, xerostomia, and dysbiosis. This practice severely compromises the predictive validity and translatability of current research, necessitating an urgent shift towards more representative models. Consequently, conclusions drawn from studies lacking OSCC-relevant models must be interpreted with extreme caution regarding their clinical applicability, as their clinical relevance is severely compromised. This underscores an urgent need for the development and widespread adoption of standardized, complex OSCC-mimicking preclinical models (e.g., irradiated rodent models, organoid-on-chip systems reflecting dysbiosis) to enhance translational success [10,11]. Second, outcome reporting remains heterogeneous, often emphasizing time-to-closure under-reporting clinically decisive endpoints (pain, oral intake, speech/swallow performance, masticatory efficiency, long-term durability, and broader patient-reported quality of life measures). To overcome this critical barrier, a consensus-driven minimum set of standardized functional and patient-reported outcomes is urgently required, ideally developed by leading professional societies or regulatory bodies, to enable meaningful comparison and accelerate patient-centric translation [31,32]. Third, manufacturing and regulatory readiness is frequently under-addressed despite being essential for translation, particularly for combination products and cell-based TEOM strategies [16,21].

For translational evaluation of oral mucosal biomaterials, an OSCC-relevant model should reproduce the key biological and functional constraints present in post-oncologic oral tissues rather than acute wound healing in healthy mucosa. Based on current head and neck oncology and regenerative medicine literature, OSCC-relevant models are defined by the presence of one or more of the following features [59,60,61,62]:

- Irradiated mucosal tissue beds. Radiotherapy exposure should replicate clinically relevant doses or fractionation schedules sufficient to induce vascular compromise, epithelial turnover impairment, fibroblast dysfunction, and fibrotic remodeling. These models better reflect delayed healing, tissue fragility, and reduced regenerative capacity observed in OSCC survivorship [59,63,64].
- Xerostomic or salivary-deficient conditions. Radiation-induced salivary gland hypofunction alters lubrication, antimicrobial defense, mucosal hydration, and epithelial repair dynamics. Xerostomic environments can be modeled through gland irradiation, pharmacologic suppression of salivation, or saliva-deficient in vitro systems incorporating altered mucin composition and reduced growth factor content [60,65].
- Altered oral microbiome or dysbiosis. Post-therapy oral tissues frequently exhibit microbial imbalance, increased pathogenic colonization, and biofilm formation that contribute to chronic inflammation and delayed healing. Relevant models incorporate defined microbial communities, infection-challenged wounds, or biofilm-associated ulcer models that simulate clinical oral dysbiosis [61,66].
- Compromised vascular or immune status. Radiochemotherapy and cancer-related systemic effects may produce local immune dysregulation, reduced angiogenic response, and impaired inflammatory resolution. Immunocompromised animal models, cytokine-altered environments, or co-culture systems incorporating immune cells can better reproduce these conditions [62,67].
- Cancerized field or residual-disease proxy conditions. The post-OSCC mucosal field may exhibit persistent molecular alterations, including chronic inflammatory signaling, stromal activation, and altered epithelial differentiation. Models incorporating premalignant epithelial changes, OSCC cell line co-cultures, or tumor–stroma interaction systems provide improved safety assessment of regenerative interventions [68,69].

An OSCC-relevant model ideally integrates multiple features (e.g., irradiation combined with xerostomia and dysbiosis), as individual factors interact synergistically to impair wound healing. Standardization of such models would substantially improve predictive validity and translational reliability of oral mucosal biomaterial research [62,70,71].

While the review thoroughly emphasizes the paramount importance of ‘oncologic-by-design’ biomaterials, a significant gap in the current evidence base is the inconsistent or absent reporting of dedicated oncologic safety evaluations within preclinical studies. The potential for regenerative cues to inadvertently support residual malignant cells or promote recurrence is a critical, often unaddressed, translational risk. Therefore, dedicated oncologic safety evaluations, including longitudinal assessments for dysplasia, recurrence surveillance, and rigorous evaluation of tumor-promoting signaling pathways (e.g., proliferation, migration, angiogenesis) in relevant OSCC cell lines and co-culture models, should be systematically incorporated into future research designs and reporting for these biomaterials.

Minimum oncologic safety parameter set (recommended for preclinical protocols):

(i) in vitro screening against OSCC-relevant epithelial lines for proliferation/migration/invasion (e.g., Ki-67/EdU, wound-healing/scratch, transwell), with and without exposure to biomaterial extracts or released cues; (ii) stromal/immune co-culture or conditioned-media paradigms reflecting a cancerized field (activated fibroblasts; inflammatory cytokine milieu); (iii) testing in an OSCC-relevant injury bed (irradiation and/or xerostomia) with predefined observation windows sufficient to detect delayed adverse remodeling; (iv) histologic surveillance for dysplasia/field-change markers and predefined criteria for adverse signaling activation (e.g., sustained pro-angiogenic/pro-proliferative pathway upregulation); (v) explicit reporting of recurrence surveillance when used in post-resection contexts.

As proof-of-feasibility for integrating oncologic readouts into biomaterial studies, postoperative implantable hydrogel platforms in oral cancer contexts have explicitly incorporated recurrence-oriented endpoints in their in vivo evaluation frameworks [20]. In reconstructive contexts, early oral mucosa equivalent grafting reports include histologic phenotype assessment and clinical surveillance (albeit in small cohorts and limited follow-up), illustrating how safety reporting can be operationalized in future trials [53,54,55].

Promising directions include “smart” hydrogels that balance adhesion and lubrication to minimize re-injury, microbiome-aware materials that provide barrier function without indiscriminate suppression of commensals, ECM-derived or ECM-mimetic scaffolds designed to bias immune responses toward constructive remodeling, and patient-specific geometries enabled by digital dentistry and additive manufacturing. Photocrosslinkable systems, such as GelMA, offer opportunities for in-situ forming constructs and bioprinting. However, intraoral photochemistry must be optimized to ensure safe light delivery and avoid cytotoxic photoinitiators [42]. Incorporation of saliva-responsive cues is also attractive, given evidence that salivary components influence wound-healing dynamics [72,73].

### 3.7. Neurosensory Considerations in Oral Mucosal Regeneration After OSCC Therapy

Neurosensory integrity, beyond epithelial and stromal repair, is an often-overlooked determinant of functional recovery after OSCC therapy. Surgical resection and adjuvant radiotherapy can disrupt sensory nerve branches (lingual, inferior alveolar, buccal, glossopharyngeal), resulting in hypoesthesia, dysesthesia, altered proprioception, and sometimes chronic neuropathic pain [74,75]. These deficits impair mastication, speech articulation, protective reflexes, and oral hygiene and may persist long after mucosal closure [75,76]. The oral epithelium also participates in bidirectional epithelial–neuronal signaling, influencing keratinocyte proliferation, barrier integrity, and wound-healing dynamics [77,78,79].

From a biomaterial standpoint, neurosensory restoration introduces tension with oncologic safety. Neurotrophic factors such as NGF, BDNF, and GDNF can promote axonal sprouting in peripheral nerve injury models [80,81], but these pathways may intersect with pro-survival, pro-migratory, and angiogenic signaling implicated in OSCC progression [27,82,83]. Consequently, most intraoral biomaterial platforms in oncologic contexts have avoided sustained delivery of potent neurogenic cues. Instead, many strategies aim for indirect neurosensory support through restoration of epithelial integrity, reduction of chronic inflammation, and normalization of ECM architecture, conditions that may permissively support endogenous nerve remodeling without exogenous neurotrophins supplementation [78,84].

Emerging approaches that may reconcile these competing priorities include ECM-mimetic scaffolds preserving neurite-guidance motifs (e.g., laminin/collagen IV), transient release of low-dose neuromodulatory peptides with rapid clearance, and mechanically compliant hydrogels that reduce aberrant mechanotransduction and secondary nerve irritation [79,85,86]. However, direct evidence linking specific biomaterial designs to safe neurosensory recovery in OSCC remains limited, highlighting a critical need for interdisciplinary research combining neuro-oncology, biomaterials science, and regenerative medicine to innovate solutions that reconcile these competing priorities; few studies quantify nerve density, sensory thresholds, or behavioral correlates of oral sensation, and oncologic follow-up is often too short to assess delayed risks [19,72]. These gaps support cautious, stepwise incorporation of neurosensory endpoints into future biomaterials studies, with explicit attention to safety in cancerized fields.

### 3.8. Risk of Bias and Limitations of the Evidence Base

Across the evidence base, risk of bias was strongly design-dependent, and the overall certainty of inference was limited by (i) small clinical cohorts outside a few trials, (ii) frequent reliance on preclinical models that do not fully replicate the post-(chemo) radiotherapy field, and (iii) inconsistent reporting of clinically decisive functional endpoints.

Overall, certainty of evidence is low-to-moderate for short-term OM symptom relief (supported by a small number of randomized studies) and low for reconstructive/long-term regenerative outcomes (dominated by case series and heterogeneous preclinical models).

Clinical evidence (symptom-oriented barrier products). The most robust human data in this review relate to mucoadhesive/barrier formulations used during active therapy, but important sources of bias remain. The MuGard trial is randomized and placebo-controlled and therefore provides the strongest internal validity among the clinical anchors (Table 2 and Table 4) [39]. However, oral mucositis outcomes often incorporate patient-reported symptoms and clinician grading; risk of bias can increase when endpoints are subjective, and outcome assessment is not fully blinded or not standardized across centers. The CAM2028/Episil study is reported as a randomized multicenter clinical trial (Table 2 and Table 4) [40], yet mucositis pain studies are intrinsically sensitive to performance and detection bias when blinding is absent or when comparators are not fully matched [87]. In contrast, radiotherapy evidence for bioadhesive barrier-forming gels includes retrospective designs (Table 2 and Table 4) [37] and therefore is at higher risk of bias due to confounding (baseline risk, supportive care co-interventions, nutritional status, and treatment intensity), selection bias, and nonuniform outcome ascertainment.

Clinical evidence (reconstruction). For reconstructive applications, reports using regenerative/bilayer dermal matrices are largely limited to clinical case series and institutional experiences (Table 2 and Table 4) [51,52]. These studies are inherently at high/critical risk of bias due to the absence of comparators, small sample sizes, and limited follow-up, which restrict confidence in comparative effectiveness and long-term durability. Future research must prioritize adequately powered randomized controlled trials to address this critical gap. Similarly, tissue-engineered oral mucosa (TEOM) evidence consists of preclinical development with early clinical grafting reports (Table 2 and Table 4) [53,54], which are typically limited by small sample size, nonrandomized designs, and short follow-up, restricting confidence in comparative effectiveness and long-term durability.

Preclinical evidence (next-generation adhesives and regenerative scaffolds). Preclinical studies underpinning wet-adhesive multifunctional hydrogels/patches and TEOM-related scaffold systems (e.g., [43,44,45,56,57]) are expanding rapidly (Table 2), but internal validity is frequently unclear because randomization, allocation concealment, blinded outcome assessment, and a priori sample size justification are often incompletely reported. This platform was evaluated in non-irradiated acute ulcer models; therefore, extrapolation to OSCC survivorship beds should be considered with caution, given the absence of irradiation, xerostomia, and dysbiosis. Among preclinical animal studies (N = 4 total), only n_1_ = 1 (≈25%) explicitly reported randomization, n_2_ = 1 (≈25%) described any blinding procedures, and n_3_ = 0 (0%) provided a sample size or power calculation; in the remaining reports, these domains were either absent or not clearly documented.

Publication and reporting biases. Common in biomaterials development, publication bias likely favors positive results, and negative/neutral formulations may be under-reported [88]. Heterogeneity in fabrication details and incomplete reporting of key material attributes (mechanics, degradation, release profiles, and sterilization) further complicate reproducibility and cross-study comparison.

Overall, the risk-of-bias profile supports cautious interpretation: clinical trials provide useful performance benchmarks for intraoral retention and symptom relief. However, given the high risk of bias across much of the clinical evidence, particularly for reconstructive and long-term regenerative claims, the certainty of inference remains low. Therefore, the field urgently requires adequately powered randomized controlled trials for all advanced biomaterials aiming for functional restoration in OSCC patients.

Table 5 consolidates the proposed research agenda, integrating OSCC-relevant model requirements, a core set of functional endpoints, technology description standards, and priority clinical trial designs.

Table 6 presents a short description of the key terms used to describe the most important findings of this review.

### 3.9. Regulatory, Scalability, and Cost Considerations

The significant and often underestimated challenges of manufacturing complexity, stringent regulatory pathways, and cost present decisive barriers to translating promising oral mucosal regenerative biomaterials, particularly advanced therapies like TEOM, into routine post-OSCC care. This systemic limitation frequently impedes the progression of even biologically sound innovations. Therefore, an ‘integrated quality-by-design’ approach must be adopted from the earliest stages of biomaterial development, explicitly incorporating considerations for manufacturability, sterilization, regulatory pathways, and health-economic endpoints to ensure successful clinical translation. Future primary research submissions should include a preliminary translational roadmap addressing these critical aspects, even if conceptual, to demonstrate awareness of these decisive barriers. Product classification strongly shapes the evidence package required for approval and adoption [89,90]. In EU settings, many current barrier products are regulated as medical devices (often Class IIa under the MDR, depending on intended use and claims), whereas in the US, they typically follow device or combination-product pathways; advanced TEOM designs align more closely with ATMP/biologic regulatory frameworks. Barrier-forming intraoral hydrogels intended primarily for protection/lubrication may be regulated as medical devices in many settings, whereas formulations delivering pharmacologically active agents (e.g., analgesics, antimicrobials, immunomodulators) may be regulated as drugs or as combination products, increasing preclinical and clinical requirements [91,92]. Acellular dermal matrices and ECM-derived scaffolds often require additional characterization of source material controls, decellularization effectiveness, residual DNA/endotoxin, and immunogenicity, while cell-based TEOM constructs generally face the most stringent requirements due to donor variability, potency testing, chain-of-custody logistics, and higher perceived risk [93].

From a scale-up perspective, a recurring translation failure point is the gap between proof-of-concept fabrication and quality-by-design manufacturing. For hydrogel systems, critical quality attributes typically include polymer molecular weight/distribution, degree of chemical modification (e.g., catechol substitution, methacrylation), crosslink density, residual monomers/photoinitiators (when applicable), viscosity/handling at point of care, and quantitative adhesion/retention performance under saliva-mimicking conditions [94,95]. For scaffolds and matrices, key attributes include porosity and pore interconnectivity, mechanical properties under wet loading, degradation kinetics, and validated sterilization that preserves structure and bioactivity. In the intraoral setting, sterilization and packaging are particularly challenging because some adhesive chemistries and bioactive components are sensitive to heat, irradiation, or ethylene oxide residuals; early selection of compatible sterilization routes, container-closure systems, and shelf-life testing must be central to translational readiness, integrated into a robust Quality-by-Design framework from the earliest stages of biomaterial development [95,96].

Cell-based TEOM approaches amplify these challenges. Manufacturing requires controlled sourcing of cells (autologous vs. allogeneic), validated identity and potency assays, sterility and mycoplasma testing, and often a cold chain. Autologous constructs introduce patient-specific production timelines that may not match surgical scheduling, while allogeneic approaches raise additional immunologic and regulatory considerations. For both, scalability is frequently limited by labor-intensive culture processes and batch-to-batch variability. As a result, TEOM translation often depends on (i) automated/closed-system bioprocessing, (ii) standardized, xeno-free reagents, (iii) defined release criteria tied to clinical performance, and (iv) delivery logistics integrated into surgical pathways [97,98].

Economic considerations are equally important in survivorship care. The adoption threshold for intraoral regenerative materials is typically determined not only by healing speed but by downstream value: reduced OM severity and opioid use, fewer treatment interruptions, faster return to oral intake, fewer wound complications and readmissions, reduced donor-site morbidity, and improved long-term function (speech/swallow/sensation). For supportive-care hydrogels, cost-effectiveness arguments may center on preventing dose reductions or breaks in radiochemotherapy and decreasing nutrition-support needs. For reconstructive scaffolds and TEOM, value propositions often relate to reducing operative time, avoiding secondary procedures, and enabling earlier functional rehabilitation, but these benefits must be demonstrated with endpoints that matter to patients and payers [53,99]. Therefore, future clinical studies should embed health-economic endpoints (resource utilization, complication costs, quality-of-life measures) alongside biological outcomes to support reimbursement and real-world implementation.

## 4. Conclusions

Bioactive hydrogels and scaffolds represent a rapidly expanding design space for oral mucosal repair after OSCC therapy. The most clinically mature hydrogel interventions remain mucoadhesive barrier-forming gels and liquids that alleviate OM pain and improve oral function during active therapy. In parallel, scaffold-based strategies, including acellular/regenerative dermal matrices for selected post-resection defects and TEOM platforms aiming to replicate epithelial stratification on supportive stromal matrices, offer potential pathways toward more durable, function-restoring reconstruction.

The next translational step in OSCC patients is not only to accelerate epithelial closure but to deliver biomaterials that (i) reliably retain and function in a wet, mechanically demanding, microbially complex environment, (ii) are explicitly designed for oncologic safety in the cancerized field, and (iii) are manufacturable, sterilizable, and scalable within realistic regulatory and cost constraints. Future research should prioritize OSCC-relevant models (especially irradiated tissue beds), standardized functional endpoints beyond closure (oral intake, durability, and neurosensory recovery), and oncologic-by-design bioactivity that avoids sustained pro-tumorigenic signaling. With these priorities, oral mucosal biomaterials can progress from symptomatic support toward clinically credible regeneration in patient care.

## Figures and Tables

**Table 1:** Design requirements for bioactive hydrogels and scaffolds for oral mucosal regeneration after OSCC therapy.

Design Requirement	Why It Matters Intraorally	OSCC/Post-Therapy-Specific Considerations	Example Measures/Endpoints
Wet retention (mucoadhesion/wet-tissue adhesion)	Maintains contact under saliva flow and shear; prevents premature loss and frictional trauma.	Adhesion must be strong yet non-traumatic on fragile, inflamed mucosa; xerostomia and altered mucins can change adhesion behavior.	Residence time; lap-shear/peel strength in saliva-mimicking media; in vivo retention time.
Mechanical compliance & durability under mastication	Withstands chewing, speech, and tongue motion without tearing or delamination.	Irradiated tissues have reduced elasticity and microvascular reserve; materials should avoid stress concentrations and secondary injury.	Wet modulus; fatigue/cyclic loading; wear/friction testing; failure mode mapping.
Barrier/lubrication with symptom relief	Reduces exposure of nerve endings, decreases pain, and supports oral intake.	Clinically decisive outcomes include pain, opioid use, and the ability to eat/drink during (chemo)radiotherapy.	Pain scales (VAS/OMDQ); oral intake; opioid consumption; treatment breaks.
Microbial control without broad microbiome harm	Limits infection/biofilm-driven inflammation and accelerates closure.	Post-therapy dysbiosis and immune compromise increase infection risk; avoids indiscriminate suppression of commensals.	Biofilm assays; microbial load; infection rate; inflammatory cytokines; dysbiosis-relevant challenge models.
Controlled, time-limited bioactivity with oncologic safety	Supports healing while minimizing pro-proliferative or pro-angiogenic signaling exposure.	Cancerized field and recurrence surveillance require “oncology-by-design” cues and explicit safety readouts.	OSCC cell line proliferation/migration assays; co-culture testing; histologic dysplasia markers; recurrence surveillance windows.
Biocompatibility & degradation matched to healing	Avoids chronic foreign-body responses and permits remodeling.	Delayed healing in irradiated/xerostomic beds may require longer persistence but must not amplify fibrosis or chronic inflammation.	Degradation kinetics; histology (fibrosis/inflammation); local toxicity; foreign-body reaction scoring.
Manufacturing, sterilization, handling, and cost	Determines real-world adoptability (clinic workflow, storage, shelf-life).	Sterilization compatibility is critical for wet-adhesive chemistries and biologics; cost constraints are high in supportive care.	Sterility assurance; shelf-life/stability; packaging; application time; cost/availability analyses.

**Table 2:** Summary of included clinical and preclinical studies.

Study (First Author, Year)	Study Type	Population/Model	*n*	Key Endpoints	Main Result (Brief)	Risk of Bias
Wei, 2025 [37]	Clinical RCT	Head and neck cancer (HNC) radiotherapy OM (OSCC/HNC-specific)	200 (ITT; 100/100)	Grade III/IV OM; pain relief; nutrition; Quality of life (QoL)	Episil reduced Grade III/IV OM (4% vs. 14%) and improved pain, nutrition, and QoL vs. control	Some concerns
Wei, 2021 [38]	Non-randomized clinical	HNC radiotherapy OM (OSCC/HNC-specific)	50 (25/25)	OM severity; PG-SGA/BMI/albumin; weight change	Barrier-forming gel associated with less severe OM and improved nutritional indicators vs. standard care.	High
Allison, 2014 [39]	Clinical RCT	HNC Chemoradiotherapy (CRT)-induced OM (OSCC/HNC-specific)	120 enrolled (78 analyzed)	Oral mucositis daily questionnaire (OMDQ) soreness Area under the curve (AUC); WHO OM score; weight; opioid use Grade III/IV OM; pain relief; nutrition; QoL	MuGard reduced patient-reported soreness AUC and WHO scores vs. control; no major safety signals.	Low
Cheng, 2018 [40]	Clinical RCT	Chemo-/radiotherapy OM (mixed oncology; non-OSCC specific)	60	Pain relief onset/duration; acceptability	CAM2028/Episil produced rapid pain reduction with an effect lasting several hours; high acceptability.	Some concerns
Galli, 2008 [41]	Clinical RCT	Oral surgical incisions (non-OSCC)	72	Wound healing; pain/discomfort; inflammation	Topical hyaluronic acid improved clinical healing parameters and reduced discomfort vs. placebo.	Some concerns
Zhang W., 2021 [43]	Animal	Acute oral mucosal wound model (non-irradiated; non-OSCC)	NR	Wound closure; histology	Photo-triggered hydrogel adhesive promoted oral mucosal wound healing in vivo.	Not assessable
Zhang Y., 2024 [44]	Animal	Acute oral ulcer model (non-irradiated; non-OSCC)	NR	Closure; barrier; microbial control	Janus hydrogel patch combined wet adhesion and a protective layer to enhance regeneration in vivo.	Not assessable
Zhang Z., 2023 [45]	Animal	Infected oral ulcer model (non-irradiated; non-OSCC)	NR	Infection control; healing; inflammation	Wet-adhesive poly(ionic liquid) patch improved healing with antibacterial/anti-inflammatory effects.	Not assessable
Cui, 2023 [46]	Animal	Acetic acid rat oral ulcers + mini-pig defect (non-irradiated; non-OSCC)	27 rats + 5 mini-pigs	Wound closure; histology; inflammatory markers	Water-responsive bioadhesive patch improved retention and accelerated healing vs. chitosan film.	Not assessable
Consorti, 2024 [51]	Non-randomized clinical	Post-resection oral cavity SCC defects (OSCC-specific)	47	Healing/complications; functional recovery	Regenerative dermal matrix used for mucosal resurfacing with acceptable healing in an observational cohort.	High
Ferri, 2025 [52]	Non-randomized clinical	Oral cavity surgical defects after SCC resection (OSCC-specific)	21	Defect closure; complications; function	Bilayer dermal matrix reconstruction was feasible with acceptable outcomes but lacks comparative data.	High
Izumi, 2000 [53]	In vitro	TEOM fabrication (serum-free) (non-OSCC)	NR	Stratification; phenotype markers	Serum-free TEOM constructs recapitulated key features of oral mucosa in vitro.	Not assessable
Izumi, 2003 [54]	Non-randomized clinical	Intraoral grafting after premalignant/cancer lesions (OSCC-relevant)	30	Graft take; epithelial persistence/maturation; histology	EVPOME showed 100% take and faster epithelial coverage/maturation vs. AlloDerm alone.	High
Lauer, 2001 [55]	Non-randomized clinical	Post-OSCC tongue release (OSCC-post-therapy)	6	Tongue mobility; sulcus formation; histologic integration	5/6 patients achieved improved tongue mobility; engineered cells integrated and normalized by 6 months.	High
Kinikoglu, 2009 [56]	In vitro	Full-thickness collagen-based oral mucosa equivalent (non-OSCC)	NR	Morphology; compartmental structure	Collagen-based full-thickness constructs reproduced epithelial and connective compartments in vitro.	Not assessable
Terada, 2012 [57]	In vitro	Chitosan–collagen TEOM scaffold (non-OSCC)	NR	Epithelial differentiation; handling/mechanics	Composite scaffold supported epithelial stratification and improved mechanical handling.	Not assessable

**Table 3:** Selected translational examples of hydrogels and scaffolds relevant to oral mucosal repair in head and neck cancer therapy and post-resection settings.

Platform/Intervention	Intervention Class	Format	Evidence Base	Population/Model	Key Translational Takeaways
MuGard (mucoadhesive hydrogel rinse)	Symptom-oriented barrier	Rinse/mucoadhesive liquid	Clinical RCT (double-blind, placebo-controlled)	HNC CRT-induced OM	Reduces patient-reported soreness and clinician OM scores; establishes a clinical benchmark for retention, tolerability, and PRO-driven benefit.
CAM2028/Episil (bioadhesive barrier-forming oral liquid)	Symptom-oriented barrier	Oral liquid that forms a protective film	Clinical RCT (randomized, multicenter; open-label)	Chemo-/radiotherapy OM (mixed oncology)	Rapid pain reduction with multi-hour duration; key benchmark for ease of use and short-term symptom relief in OM care pathways.
Barrier-forming gel (Episil) in head & neck radiotherapy	Symptom-oriented barrier	Spray/oral liquid barrier	Clinical RCT + retrospective study	HNC radiotherapy OM	Associated with reduced severe OM and improved nutrition/QoL metrics; primarily supportive care (not a regenerative matrix).
Hyaluronic acid formulations	Regenerative/bioactive hydrogel	Topical gel	Pilot clinical RCT (placebo-controlled)	Oral surgical incisions (non-OSCC)	Improves clinical healing parameters; extrapolation to OSCC survivorship beds requires caution when irradiation/xerostomia are absent.
Regenerative/acellular dermal matrices (incl. bilayer matrices)	Regenerative matrix/scaffold	Sheet-like dermal substitute	Clinical case series/institutional experience	Post-resection oral cavity SCC defects	Feasible for mucosal resurfacing and donor-site sparing; needs comparative trials, standardized fixation, and long-term durability/safety reporting.
TEOM/Ex vivo produced oral mucosa equivalent (EVPOME) (keratinocytes on dermal matrix)	TEOM/regenerative scaffold	Cell-based mucosal substitute	Early clinical reports (comparative study + small cohorts)	Intraoral grafting/lining reconstruction (OSCC-relevant contexts)	High take rates and epithelial maturation reported; translation constrained by manufacturing complexity, cost, and regulatory burden.
Wet-adhesive multifunctional hydrogel patches (e.g., S-nitrosylation, poly(ionic liquid), water-responsive, Janus patches)	Regenerative hydrogel patch	Preformed adhesive patch	Preclinical (animal)	Non-irradiated acute oral ulcer/defect models	Demonstrate strong wet adhesion plus antimicrobial/anti-inflammatory functions and faster closure; OSCC extrapolation should be cautious without irradiated/xerostomic models.

**Table 4:** Qualitative risk-of-bias considerations for key clinical studies.

Study	Design	Main RoB Concerns
Episil in HNC RT (Wei, 2025) [37]	RCT (multicenter)	Randomization reported; blinding unclear/limited; subjective pain outcomes may introduce performance/detection bias.
Barrier-forming gel in HNC RT (Wei, 2021) [38]	Retrospective cohort	High confounding risk (supportive care/nutrition/treatment intensity); selection bias; outcome ascertainment not standardized.
MuGard (Allison, 2014) [39]	RCT (double-blind, placebo-controlled)	Low concern for allocation concealment/blinding; main concern is attrition/per-protocol efficacy analysis (78/120 analyzed) and subjective endpoints.
CAM2028/Episil (Cheng, 2018) [40]	RCT (randomized; open-label; positive-controlled)	No blinding → performance/detection bias for pain outcomes; comparators not fully matched; short-term/single-use endpoint focus.
Dermal matrices (Consorti, 2024 [51]; Ferri, 2025) [52]	Case series/retrospective	No comparator; selection bias; small samples; heterogeneous defects and follow-up; selective reporting of long-term functional endpoints possible.
TEOM/EVPOME (Izumi, 2003) [54]	Non-randomized comparative study	Group allocation by disease stage (non-random); no blinding; small sample; limited follow-up → high risk of bias.
Tissue-engineered mucosa graft (Lauer, 2001) [55]	Small clinical study (*n* = 6)	No comparator; small sample; potential selection and reporting bias; limited generalizability.

**Table 5:** Translational roadmap for oral mucosal biomaterials after OSCC therapy.

	Bench/Design	Preclinical OSCC-Relevant Models	Early-Phase Trials	Comparative Trials
Model requirements	Define target use-case (OM vs. resection bed) Simulate saliva + shear in vitro Include biofilm/dysbiosis challenge where feasible	Irradiation (clinically relevant dosing) Xerostomia/salivary deficiency Dysbiosis/biofilm challenge Cancerized field proxies/tumor–stroma interactions	Feasibility + safety in HNC/OSCC cohorts Stratify by RT dose/xerostomia severity Monitor local toxicity and healing complications	Standardized OSCC/HNC patient populations Long-term follow-up (function + safety) Harmonized supportive care comparators
Core functional endpoints	Wet adhesion/retention under saliva Barrier integrity + friction/lubrication Cytocompatibility and degradation	Durability under mastication/tongue motion Histology: epithelial stratification + remodeling Infection/biofilm metrics (Where possible) neurosensory proxies	Pain, oral intake, speech/swallow Opioid use and treatment breaks Device retention/handling QoL and adverse events	Comparative effectiveness vs. benchmarks Durability and late breakdown Neurosensory outcomes Patient-reported QoL and health-economic endpoints
Technology description requirements	Mechanics (wet modulus/fatigue) Degradation + byproducts Sterilization/storage stability Handling/application time Cost/availability assumptions	Batch-to-batch QC and reproducibility Quantitative adhesion metrics in vivo Standardized reporting of fabrication and dosing	SOPs for use in clinic Training/handling requirements Preliminary cost/resource utilization capture	Manufacturing scale-up plan Regulatory dossier alignment Reimbursement-relevant outcomes and supply chain feasibility
Priority clinical research directions	Benchmark against symptom-oriented barriers Define minimal performance specs for translation	Comparative studies in OSCC-relevant beds Reconstruction trials with ADM/TEOM in irradiated fields Oncologic safety readouts embedded in protocols	Early-phase trials: safety, retention, acceptability Reconstruction feasibility (ADM/TEOM) Prospective oncologic surveillance plans	Multicenter comparative RCTs vs. barrier benchmarks Long-term follow-up for oncologic safety and function Implementation studies in real-world care pathways

**Table 6:** Mini-glossary of key platform terms used in this review.

Term	Definition
Barrier-forming gel/oral liquid	Topical formulation that forms a protective film over the mucosa to reduce friction and pain and to protect ulcers during OM; typically intended for short-term symptom mitigation rather than structural regeneration.
Mucoadhesive rinse	Liquid oral rinse designed to adhere to mucosa via mucoadhesive interactions, prolonging residence time and providing lubrication/barrier effects (e.g., for OM symptom control).
Wet-adhesive patch	Patch with wet-tissue adhesion designed for intraoral retention under saliva and shear; often engineered with catechol/ionic/dynamic covalent chemistries and may incorporate antimicrobial or bioactive payloads.
Scaffold (acellular matrix vs. synthetic scaffold)	A 3D structure providing mechanical support and guidance for tissue repair. Acellular matrices preserve native ECM architecture/cues; synthetic scaffolds provide tunable mechanics/degradation and often require bioactive ligands.
TEOM	Tissue-engineered oral mucosa: a cell-based mucosal substitute (typically keratinocytes ± stromal cells on a scaffold) designed to replicate stratified oral epithelium for lining reconstruction.

## Data Availability

The data presented in this study is available on request from the corresponding author.

## Supplementary Materials

The following supporting information can be downloaded at: https://www.mdpi.com/article/10.3390/medicina62030558/s1, Checklist item (PRISMA 2020).

## Abbreviations

The following abbreviations are used in this manuscript:

ADM	Acellular dermal matrix
ECM	Extracellular matrix
GelMA	Gelatin methacryloyl
HNC	Head and neck cancer
OM	Oral mucositis
OSCC	Oral squamous cell carcinoma
TEOM	Tissue-engineered oral mucosa
