# Supplementary material for: Bioactive Hydrogels and Scaffolds for Oral Mucosal Regeneration After Oral Squamous Cell Carcinoma Therapy: A Comprehensive Review
Source: Medicina (Kaunas). 2026 Mar 17;62(3):558. doi: 10.3390/medicina62030558 (PMC13027492; doi:10.3390/medicina62030558)
Supplement: Supplementary file 1 [file medicina-62-00558-s001.zip › medicina-4168688-supplementary.pdf]

Manuscript: Bioactive Hydrogels and Scaffolds for Oral Mucosal Regeneration after Oral Squamous Cell Carcinoma Therapy: A Comprehensive Review

Section/Topic	Item	Checklist item (PRISMA 2020)	Reported?	Location in manuscript	Notes
Title	1	Identify the report as a systematic review.	Partial	p.1 (title page)	Title states 'Review'/'Comprehensive Review' but not explicitly 'systematic review'.
Abstract	2	Provide a structured summary of the review (per PRISMA for Abstracts).	Partial	p.2 (Abstract)	Abstract is unstructured; does not report search dates or number of included studies.
Introduction	3	Describe the rationale for the review in the context of existing knowledge.	Yes	p.3–4 (Introduction)	
Introduction	4	State the review objectives/questions.	Yes	p.4 (end of Introduction; 'This review first delineates…')	
Methods	5	Specify inclusion and exclusion criteria; describe how studies were grouped for syntheses.	Yes	p.6 (Section 2.4 Eligibility criteria)	
Methods	6	Specify information sources (databases/registers/other) and date last searched.	Partial	p.5 (Section 2.2); search dates referenced in Supplementary Table S1 (mentioned p.6)	Databases listed; exact search dates not reported in main text.
Methods	7	Present full search strategy for each source (including limits/filters).	Partial	p.5–6 (Section 2.3); full strategies in Supplementary Table S1 (mentioned p.6)	Main text provides concept blocks + example terms, but not full reproducible strategies.
Methods	8	Describe selection process (screening/eligibility), number of reviewers, independence, and any automation tools.	Partial	p.7 (Section 2.5); Figure 1 p.5	Two reviewers reported; independence and automation tools not specified.
Methods	9	Describe data collection process, number of reviewers, independence, how disagreements handled, and any automation; note how missing data were obtained.	Partial	p.7 (Section 2.5)	Standardized charting described; reviewer roles/independence and contact for missing info not described.
Methods	10a	List and define all outcomes for which data were sought.	Partial	p.7 (Section 2.5, bullet: 'Outcome measures…')	Outcomes listed broadly but not fully defined (e.g., primary vs secondary) and no handling of missing outcome data described.
Methods	10b	List and define other variables for which data were sought; describe assumptions about missing/unclear	Partial	p.7 (Section 2.5, bullet list on	Variables listed; assumptions about missing/unclear

		information.		material/platform/model variables)	information not described.
Methods	11	Describe methods used to assess risk of bias, number of reviewers, independence, and any automation tools.	Partial	p.7 (Section 2.5, RoB 2.0/ROBINS-I; qualitative appraisal for preclinical)	Tools named; reviewer process not specified; no results table for RoB.
Methods	12	Specify effect measure(s) for each outcome (e.g., risk ratio, mean difference).	N/A	N/A (no quantitative synthesis/meta-analysis)	
Methods	13a	Describe how studies were selected/assigned to each synthesis.	Partial	p.7–8 (Section 2.6; stratification by material class, format, intended function)	Taxonomy described; not explicit mapping of each included study to each synthesis.
Methods	13b	Describe methods used to prepare data for presentation/synthesis (e.g., conversions, handling missing stats).	No	Not reported	No data-preparation methods described (likely because narrative synthesis).
Methods	13c	Describe methods used to tabulate/visually display results.	Partial	p.9 (Table 1); p.13–14 (Table 2); narrative synthesis p.8–20	Tables used but display methods not described in Methods.
Methods	13d	Describe methods used to synthesize results; rationale for chosen synthesis; software if used.	Yes	p.7–8 (Section 2.6 Evidence synthesis and analytical framework)	Narrative framework-based synthesis; meta-analysis not feasible.
Methods	13e	Describe methods used to explore heterogeneity (e.g., subgroup analysis/meta-regression).	N/A	N/A (no meta-analysis; qualitative synthesis)	
Methods	13f	Describe sensitivity analyses performed.	N/A	N/A (no quantitative synthesis)	
Methods	14	Describe methods used to assess risk of bias due to missing results (reporting bias).	No	Not reported (qualitative discussion only)	Reporting/publication bias is discussed (p.19) but no formal methods described.
Methods	15	Describe methods used to assess certainty/confidence in the body of evidence (e.g., GRADE).	No	Not reported	No certainty framework reported.
Results	16a	Describe search and selection results, including numbers screened/included; provide flow diagram.	Yes	p.5 (Figure 1 PRISMA flow diagram); p.4 and p.7 refer to Figure 1	Flow diagram reports n=20 included.
Results	16b	Cite studies that were excluded after full-text assessment and give reasons.	Partial	p.5 (Figure 1 gives counts); no study-level exclusion list	PRISMA asks for citations + reasons; manuscript provides only counts.
Results	17	Cite included studies and present their characteristics.	Partial	p.13–14 (Table 2 selected examples); narrative throughout Results p.8–20	Table 2 is selective; does not list all included studies (n=20).
Results	18	Present risk of bias assessments for each included study.	Partial	p.18–19 (Section 3.8 Risk of bias and limitations)	Narrative RoB discussion; no per-study RoB table/figure.
Results	19	For all outcomes, present results for individual studies	Partial	Narrative Results p.8–20;	No standardized per-study

		(summary statistics/effect estimates) and syntheses.		Table 2 p.14	quantitative results (consistent with qualitative review).
Results	20a	For each synthesis, summarize contributing studies and their risk of bias.	Partial	Results sections 3.1–3.9 (p.8–20)	Syntheses described narratively; contributing-study lists and RoB summaries not explicit.
Results	20b	If meta-analysis done, present pooled estimates and heterogeneity.	N/A	N/A (no meta-analysis)	
Results	20c	If heterogeneity investigated, present those results.	N/A	N/A	
Results	20d	If sensitivity analyses done, present those results.	N/A	N/A	
Results	21	Present assessments of risk of bias due to missing results (reporting biases) for each synthesis.	Partial	p.19 (Section 3.8 Publication and reporting biases)	Qualitative statement; no synthesis-level assessment.
Results	22	Present assessments of certainty/confidence in the body of evidence for each outcome.	No	Not reported	
Discussion	23a	Provide a general interpretation of results in context of other evidence.	Yes	p.8–21 (Results synthesis + Conclusions)	Interpretation integrated across Results and Conclusions.
Discussion	23b	Discuss limitations of the evidence included in the review.	Yes	p.18–19 (Section 3.8)	Addresses design-dependent bias, model limitations, reporting issues.
Discussion	23c	Discuss limitations of the review processes used.	Partial	p.7 (risk-of-bias tool application constraints for preclinical); p.18–19 (bias discussion)	No dedicated section on review-process limitations (e.g., search restrictions, screening/data extraction verification).
Discussion	23d	Discuss implications for practice, policy, and future research.	Yes	p.2 (Abstract); p.19–21 (Sections 3.9 and Conclusions)	Translational priorities and future directions stated.
Other information	24a	Provide registration information (register name and ID) or state not registered.	No	Not reported	
Other information	24b	Indicate where protocol can be accessed or state none.	No	Not reported	
Other information	24c	Describe and explain amendments to registration/protocol.	N/A	N/A (no registration/protocol reported)	
Other information	25	Describe sources of support/funding and role of funders.	Yes	p.21 (Funding statement)	No external funding stated.
Other information	26	Declare competing interests.	Yes	p.21 (Conflicts of Interest)	
Other information	27	Report availability of data, code, and other materials.	Yes	p.21 (Data Availability Statement)	Data available on request.

From: Page MJ, McKenzie JE, Bossuyt PM, Boutron I, Hoffmann TC, Mulrow CD, et al. The PRISMA 2020 statement: an updated guideline for reporting systematic reviews. BMJ 2021;372:n71. doi: 10.1136/bmj.n71. This work is licensed under CC BY 4.0. To view a copy of this license, visit https://creativecommons.org/licenses/by/4.0/
